# Decreased serum levels of angiotensin converting enzyme (ACE)2 and enhanced cytokine levels with severity of COVID-19: normalisation upon disease recovery

**DOI:** 10.1016/j.heliyon.2022.e08957

**Published:** 2022-02-16

**Authors:** Esmaeil Mortaz, Hamidreza Jamaati, Neda Dalil Roofchayee, Hakime Sheikhzade, Maryam Mirenayat, Mohsen Sadeghi, Somayeh Lookzadeh, Neda K. Dezfuli, Gert Folkerts, Sharon Mumby, Johan Garssen, Ian M. Adcock

**Affiliations:** a Department of Immunology, School of Medicine, Shahid Beheshti University of Medical Sciences, Tehran, Iran; b Clinical Tuberculosis and Epidemiology Research Center, National Research Institute of Tuberculosis and Lung Diseases (NRITLD), Shahid Beheshti University of Medical Sciences, Tehran, Iran; c Chronic Respiratory Diseases Research Center, National Research Institute of Tuberculosis and Lung Diseases (NRITLD), Shahid Beheshti University of Medical Sciences, Tehran, Iran; d Department of Immunology and Laboratory Sciences, School of Allied Medical Sciences, Dezful University of Medical Sciences, Dezful, Iran; e Division of Pharmacology, Faculty of Science, Utrecht Institute for Pharmaceutical Sciences, Utrecht University, Utrecht, Netherlands; f National Heart and Lung Institute, Imperial College London and the NIHR Imperial Biomedical Research Centre, London, UK; g Center of Excellence Nutricia Research, Utrecht, Netherlands

**Keywords:** COVID-19, Remdesivir, Dexamethasone, Cytokines, IL-6

## Abstract

**Background:**

Severe acute respiratory syndrome coronavirus 2 (SARS-CoV-2) causes coronavirus disease 2019 (COVID-19). Circulating soluble angiotensin-converting enzyme (sACE2)2, the main receptor for SARS-CoV-2, together with components of the renin-angiotensin system promote infection and disease severity.

**Objective:**

This pilot study followed the time-course of sACE2 levels in relation to systemic cytokines in severe and moderate COVID-19 patients treated with remdesivir/dexamethasone in combination.

**Methods:**

Peripheral blood was obtained upon admission from 30 patients (12 with moderate disease and 18 with severe disease) and 14 patients with PCR-confirmed mild COVID-19. Severe and moderate patients were treated with remdesivir (200mg/first day and 100mg/day for the remaining days) and dexamethasone (100mg/day). 6 healthy control subjects (HC) were also enrolled. Serum interleukin (IL)-6 and IL-8 and sACE2 levels were measured by ELISA at baseline and during treatment in severe and moderate patients and at baseline in mild and HCs.

**Results:**

Baseline sACE2 levels were lower in severe (p = 0.0005) and moderate (p = 0.0022) patients than in patients with mild COVID-19 and in HC (p = 0.0023 and p = 0.0012 respectively). Treatment significantly increased sACE2 levels in patients with moderate disease (p = 0.0156) but only 50% of patients with severe disease showed enhanced levels compared to baseline. Systemic IL-6 and IL-8 levels were higher in all patient groups compared with HC and were not significantly affected over time or by remdesivir/dexamethasone treatment for 5 days.

**Conclusion:**

Serum sACE2 levels increase in severe COVID-19 patients as they recover over time whilst circulating cytokines are unaffected. Future studies should link these results to clinical outcomes.

## 1 Introduction

Severe acute respiratory syndrome virus 2 (SARS-CoV-2) causes a respiratory disease that led to the fatal Coronavirus disease 2019 (COVID-19) pandemic [1]. COVID-19 is heterogeneous and manifests clinically with fever, cough, muscle pain, fatigue, loss of taste and smell, diarrhoea and pneumonia and may result in death in susceptible subjects [2, 3, 4]. Patients with poor prognostic features upon hospital admission frequently encounter complications with significant mortality associated with acute respiratory distress syndrome (ARDS), multi-organ failure and blood clots [5].

The renin–angiotensin system (RAS) maintains blood pressure and electrolyte balance in the body and has been implicated in the pathogenesis of ARDS also [6]. RAS operates via two axes: the classic angiotensin converting enzyme (ACE)/Angiotensin (Ang) II/Ang II type 1 (AT_1_) receptor axis and the non-classical ACE2/Ang 1–7/Mas receptor (MasR) axis. These two pathways have opposing functions: whilst the former is associated with impairment of respiratory conditions the latter plays a protective role in ARDS [7]. Thus, determining the role of RAS in the pathogenesis of COVID-19 in controlling, monitoring and management of COVID-19 is essential.

ACE2 is a carboxypeptidase and type I transmembrane protein with an extracellular N-terminal domain containing the active site [8] and is recognised as a receptor for SARS-CoV-2 infection [9]. ACE2 is secreted into the systemic circulation as an enzymatically active ectodomain also [8]. After binding to ACE2 and entry of the SARS-CoV-2 to the target cells, shedding of host ACE2 receptors occurs that may disrupt RAS tissue homeostasis leading to important implications for COVID-19 severity [10, 11, 12]. Interaction of RAS-associated proteins with circulating soluble ACE2 (sACE2) is essential for SARS-CoV-2-mediated entry [13]. Furthermore, injection of recombinant ACE2 has a potential therapeutic role in treating patients infected with SARS-CoV-2 [12, 13, 14].

We hypothesised that increased sACE2 levels may protect against severe COVID-19 by blocking viral entry to target cells [15, 16, 17]. Thus, we investigated the levels of sACE2 in mild, moderate and severe COVID-19 patients and assessed whether an anti-viral treatment together with dexamethasone affected sACE2 levels.

## 2 Materials and methods

### 2.1 Patient sample collection

Thirty confirmed COVID-19 patients including 18 patients with severe disease, 12 with moderate disease and 14 subjects with mild disease who did not require hospitalization were enrolled into the study upon admission to the Masih Daneshvari Hospital of Shahid Beheshti Medical University (Tehran-Iran) between Jan. 10^th^- Feb. 5^th^, 2021. Six healthy control subjects (HC) were enrolled as controls. All patients were diagnosed according to World Health Organization (WHO) interim [18, 19]. All COVID-19 patients were confirmed as polymerase chain reaction (PCR) positive using specific primers for SARS-CoV-2 in their nasopharyngeal samples.

Severe COVID-19 disease was confirmed by the presence of at least one of the following: respiratory rate >30/min; blood oxygen saturation ≤90% on room air; ratio of partial pressure of oxygen in arterial blood to the inspired oxygen fraction (PaO2FiO2) <300 and lung infiltrates present on >50% of the lung fields. In contrast, patients with moderate disease were characterized with respiratory rate ≥24 and SpO2 ≤93% on room air and mild disease was characterized by upper respiratory tract symptoms without shortness of breath or hypoxia [20]. The study was approved by the institutional ethics board of the Masih Daneshvari Hospital (Ethics number SBMU.NRITLD.REC.1399.226).

### 2.2 Data collection

The clinical records of patients were interpreted by the research team of the Department of Critical Care Medicine, Masih Daneshvari Hospital of Shahid Beheshti University. Clinical, laboratory, and radiological properties and treatments and outcomes data were collated from electronic medical records. The information recorded included demographic data, medical history, underlying comorbidities, symptoms, signs, laboratory findings; chest computed tomographic (CT) scans, and treatment measures. The antiviral treatment in this study for the moderate and severe patients was remdesivir (200mg/first day and 100mg/day for the 4 days) with dexamethasone (100mg/day) as previously described [21]. Besides in all severe patients were treated with methylprednisolone (125 mg–500 mg) for three days and for rest with dexamethasone (8–16 mg for 5 days) and the moderate patient only got dexamethasone as the same as severe patients. In addition, all patients with severe disease received a single dose of Actemra (anti-IL-6, 8 mg/kg) upon admission to hospital as recommended [22].

### 2.3 Laboratory examination of blood samples

Blood (3ml in tubes without anticoagulants) was obtained at baseline and 5 days after initiation of antiviral treatment and other supportive therapy. The erythrocyte sedimentation rate (ESR) was determined in citrate-treated whole blood samples. For total blood samples containing, anti-coagulant EDTA (3ml) were obtained from all participants upon admission and 7 days after antiviral therapy.

Serum samples were separated by centrifugation at 1200 x g for 5 min. Serum biochemical tests (including kidney and liver function tests including phosphocreatine kinase (CPK), lactate dehydrogenase (LDH), electrolytes, C reactive protein (CRP) and myocardial enzymes were obtained.

### 2.4 Analysis of serum sACE2, IL-6 and IL-8

Serum sACE2 was measured using an ELISA kit (Mybiosource, San Diego, CA) according to the following Manufactures' instructions. The human ACE2 ELISA Kit has a detection range of 62.5–4000 pg/ml. Circulating levels of IL-6 (a detection range of 9.4–600 pg/ml) (R&D Biosystem) and IL-8 (OptEIA™ assay, detection ≥2 pg/mL) (BD Bioscience) were evaluated in the serum of participants before and after treatment according to the following manufacturer's instructions.

### 2.5 Statistical analysis

Analysis of data was performed using the SPSS program version 16.0 (SPSS, Inc. Chicago, USA) and GraphPad Prism software (version 8 Graph Pad Software, Inc.). A non-parametric Mann-Whitney U test (Median, 5–95% percentile was used for the variables not normally distributed (Table 2). An unpaired t-student test (Mean, 95% confidence intervals (CI) was used for parametric variables. Normality was assessed by the Kolmogorov–Smirnov test (Table 1). A Fishers's exact test was used for calculating the male/female ratio in Table 1. A paired Wilcoxon test was used for the before and after treatment comparison in the severe and moderate patients. p < 0.05 was considered as statistically significant.

**Table 1:** Demographic characteristics of participants before (BT) and after treatment (AT) with remdesivir and dexamethasone.

	Sev, ICU (n = 18)	Mod, non-ICU (n = 12)	Mi (n = 14)	HC (n = 6)	P value Sev vs. Mod	P value patients vs. HC	P value∗ (Male/Female ratio)
Age, years	59.88 ± 3.167	53.50 ± 4.424	49.86 ± 6.35	46.50 ± 6.158	0.2391	0.1043	
Male, N (%)	11 (61.2)	5 (41.6)	7 (50)	6 (100)			0.4572
Female, N (%)	7 (38.8)	7 (58.4)	7 (50)	2 (33.33)			
RR/BPM	20.17 ± 1.138, n = 6	19.86 ± 1.033, n = 7			0.8438		
O_2_ saturation (%)	81.67 ± 2.060, n = 6	90.86 ± 0.7693, n = 7			**<0.001**		
CT scan (%)	45.00 ± 3.162, n = 6	19.29 ± 3.168, n = 7			**<0.0001**		
APACHE II score	12.67 ± 1.202, n = 6	4.571 ± 0.6117, n = 7			**<0.0001**		

Values were presented as mean ± SEM.

**Abbreviations used:** APACHE: Acute Physiology and Chronic Health Evaluation, AT; after treatment, BT; before treatment, CT: computerised tomography, Mod; Moderate, Mi: Mild, RR: Respiratory rate, BPM: breaths per minute; Sev: Severe.

∗ Male/Female ratio was calculated with Fisher's exact test across the 2 studied groups (severe, moderate).

## 3 Results

### 3.1 Demographic and clinical characteristics of patients and healthy controls

Basic demographic and clinical characteristics of the participants are shown in Table 1. The mean age of the COVID-19 patients was not significantly different from that of the healthy controls (57.1 ± 2.6 versus 46.5 ± 6.2 years, p = 0.1043) but gender was different due to all the control subjects being male. Table 1 also shows the respiratory rate (RR), CT values and SpO2 and APACHEII of subjects at baseline.

Serum ESR (p = 0.0013), CRP (p = 0.0067), LDH (p = 0.0004) and CPK (p = 0.0077) levels were higher in severe COVID-19 patients at baseline than in mild patients whilst baseline levels of all but CPK (p = 0.1193) were also higher in moderate patients compared with mild patients (Table 2). These were not measure in healthy subjects.

**Table 2:** Biochemical features of patients before (BT) and after treatment (AT) with remdesivir and dexamethasone.

		ESR (mm/hr)	CRP (mg/l)	LDH (U/L)	CPK (U/L)
Sev, ICU (n = 18)	BT	57.71 ± 7.19, (n = 14)	45.64 ± 6.25, (n = 14)	747.2 ± 63.42, (n = 15)	109.5 ± 19.11, (n = 13)
	AT	27.89 ± 7.75, (n = 9)	22.18 ± 6.17, (n = 11)	1179 ± 223.6, (n = 6)	64.67 ± 14.52, (n = 3)
Mod, non-ICU (n = 12)	BT	46.92 ± 7.81, (n = 12)	60.55 ± 5.80, (n = 11)	658.1 ± 66.52, (n = 11)	94.00 ± 36.27, (n = 8)
	AT	28.22 ± 6.68, (n = 9)	16.10 ± 3.95, (n = 10)	527.0 ± 10.15, (n = 3)	37.00 ± 9.07, (n = 3)
Mild (n = 14)		14.83 ± 2.66	15.83 ± 1.40	311.0 ± 18.01	22.83 ± 3.70
P value BT (Mod) vs. BT (sev)		0.3189	0.1013	0.35	0.6828
P value AT (Mod) vs. AT (Sev)		0.9744	0.4271	0.0866	0.1814
P value BT (Sev) vs. Mild		**0.0013**	**0.0067**	**0.0004**	**0.0077**
P value AT (Sev) vs. Mild		0.2078	0.4686	**0.0031**	**0.0065**
P value BT (Mod) vs. Mild		**0.0122**	**<0.0001**	**0.0019**	**0.1193**
P value AT (Mod) vs. Mild		0.1418	0.9604	**<0.0001**	0.1214
P value BT (Sev) vs. AT (Sev)∗		**0.0273**	**0.0049**	0.1563	0.2500
P value BT (Mod) vs. AT (Mod)∗		**0.0195**	**0.002**	0.2500	0.1112

Values were presented as mean ± SEM.

**Abbreviations used:** CPK: Creatine phosphokinase, CRP: C-reactive protein, ESR: Erythrocyte sedimentation rate, LDH: Lactate dehydrogenase, Mod; Moderate, Mi: Mild, Sev: Severe.

∗ Comparisons of data between the groups were performed using Wilcoxon's paired test.

Over time, the serum levels of ESR (p = 0.0273) and CRP (p = 0.0049) were significantly reduced in severe patients. Similarly, CRP (p = 0.002) and ESR (p = 0.0195) levels were significantly reduced in patients with moderate disease (Table 2). This coincided with the introduction of remdesivir and dexamethasone therapy for 5–7 days. Overall, the serum levels of ESR and CRP in severe patients after 5–7 days treatment with remdesivir and dexamethasone were similar to those seen in mild patients whilst the level of LDH (p = 0.0031) and CPK (p = 0.0065) remained significantly different from those in mild subjects (Table 2). In comparison, only the serum levels of LDH in moderate COVID-19 patients after 5–7 days were significantly different (p < 0.0001) from the levels seen in subjects with mild disease (Table 2). There was no significant difference in LDH, CRP, ESR and CPK levels between severe and moderate patients (Table 2).

### 3.2 sACE2 levels at baseline and over time

**Figure 1:**
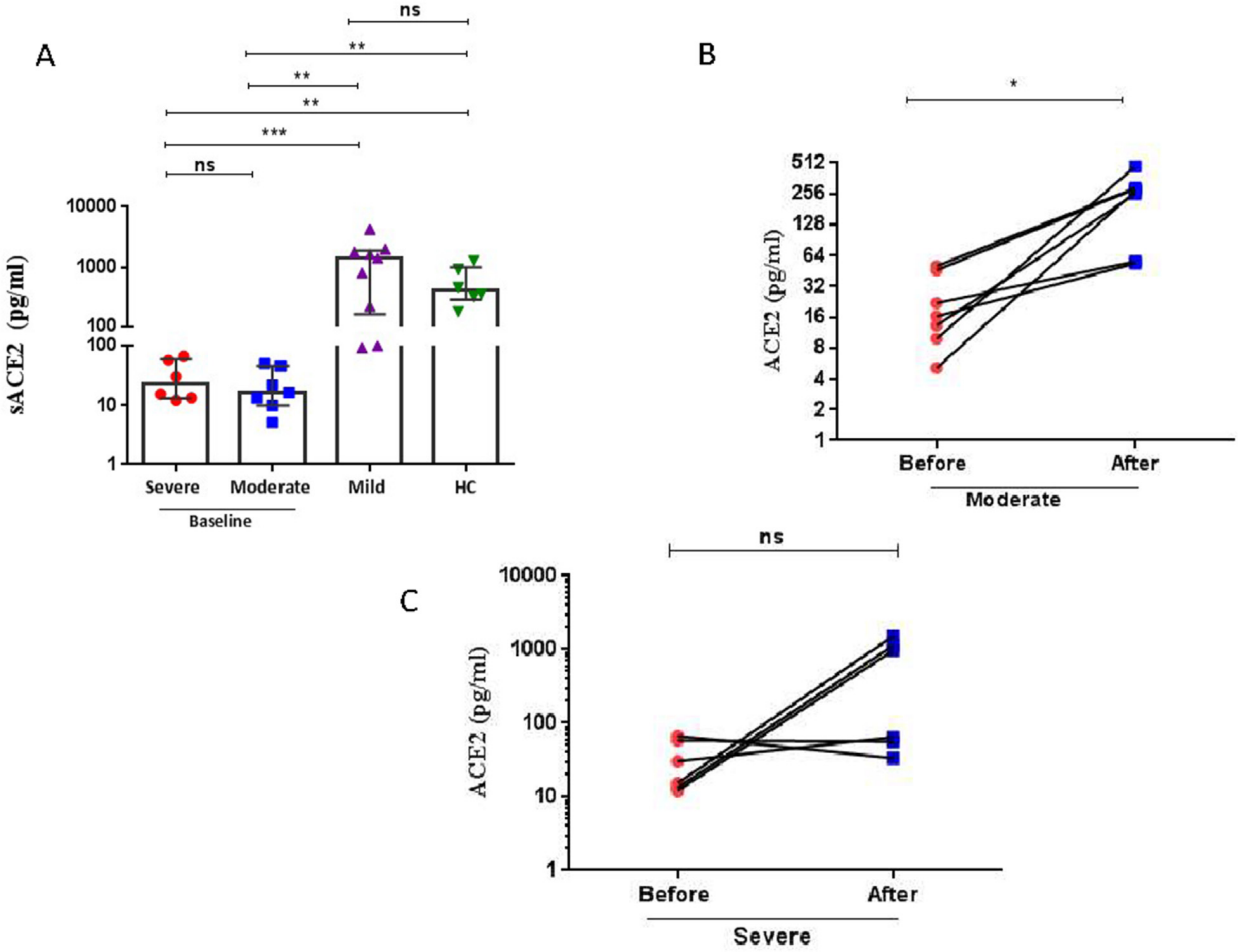
Serum levels of soluble angiotensin converting enzyme (ACE)2 in COVID-19 patients. (A) Dot blots of individual values and median (5–95% percentiles) of serum sACE2 levels before remdisivir and dexamethasone therapy in severe and moderate COVID-19 patients compared with levels in mild patients and healthy control (HC) subjects. (B) The comparison of sACE2 levels before and after 5–7 days remdisivir and dexamethasone therapy in moderate COVID-19 patients. (C) The comparison of sACE2 levels before and after 5–7 days remdisivir and dexamethasone therapy in severe COVID-19 patients. A Mann-Whitney non-parametric test was used to analyse data between groups and a paired Wilcoxon non-parametric test was used for the before and after treatment comparison. ∗p < 0.05, ∗∗p < 0.01, ∗∗∗p < 0.001, ns; nonsignificant.

Baseline sACE2 levels in severe patients were significantly lower than observed in patients with mild COVID-19 (p = 0.0005) or HC (p = 0.0022) (Figure 1A, Tables 3 & 4). Similarly, baseline sACE2 levels in moderate COVID-19 patients were also significantly lower than observed in patients with mild disease (p = 0.0023) or HC (p = 0.0012) (Figure 1A, Tables 3 & 4). No difference in baseline sACE2 level was observed between HC and mild COVID-19 patients (Figure 1A, Tables 3 & 4). There was a significant difference in sACE2 levels between the baseline and after treatment in moderate patients (P = 0.0156) (Figure 1B, Tables 3 & 4). No difference in sACE2 levels was observed between the baseline and after treatment in severe patients (P = 0.1563) (Figure 1C, Tables 3 & 4) due to only 50% of subjects responding. Induction of sACE2 over 5–7 days in severe and moderate COVID-19 patients resulted in similar sACE2 levels to those seen in baseline levels in subjects with mild COVID-19 and HC (Figure 1A, Tables 3 & 4).

**Table 3:** Serum ACE2 and cytokine levels before (BT) and after (AT) remdesivir and dexamethasone treatment.

	Severe, ICU		Moderate, non-ICU		Mild	HC (n = 6)
	BT	AT	BT	AT		
sACE2 (pg/ml)	22.8 (12.1–66.9) n = 6	514.7 (33.6–1517) n = 6	16.32 (5.1–50.4) n = 7	270.4 (53.5–473.3) n = 7	1114 (64–4388) n = 10	404 (180–1290)
IL-6 (pg/ml)	17.74 (0.13–288.6) (n = 11)	23.29 (12.04–224.2) (n = 11)	14.38 (2.113–58.48) (n = 8)	14.93 (6.74–20.29) (n = 8)	10 (4.7–34.2) n = 13	2.25 (1.2–3.1)
IL--8 (pg/ml)	2.12 (0.18–3.92) (n = 8)	4.97 (0.17–21.88) (n = 7)	0.95 (0.15–32.72) (n = 13)	1.99 (0.1–10.38) (n = 11)	1.32 (0.50–10.38) n = 13	0.22 (0.1–0.45)

Values were presented as Median and 5–95% percentile.

**Abbreviations used**: HC; healthy control subjects.

**Table 4:** P values for comparisons between serum ACE2 and cytokine levels before (BT) and after (AT) remdesivir and dexamethasone treatment.

	P values		
	sACE2 (pg/ml)	IL-6 (pg/ml)	IL--8 (pg/ml)
BT (Moderate) vs. BT (Severe)	0.4685	0.6574	0.9155
BT (Moderate) vs. HC	**0.0012**	**0.008**	**0.0047**
BT (Severe) vs. HC	**0.0022**	**0.036**	**0.008**
BT (Moderate) vs. Mild	**0.0023**	0.9015	0.8010
BT (Severe) vs. Mild	**0.0005**	0.7649	0.5002
Mild vs. HC	0.3884	**<0.0001**	**<0.0001**
P value BT (Moderate) vs. AT (Moderate)∗	**0.0156**	0.6406	0.4131
P value BT (Severe) vs. AT (Severe)∗	0.1563	0.7002	0.0781

Comparisons of data between the groups were performed using the Mann-Whitney U test.

∗ Comparisons of treatment effects within groups were performed using the paired Wilcoxon test.

### 3.3 Serum cytokines levels at baseline and over time

**Figure 2:**
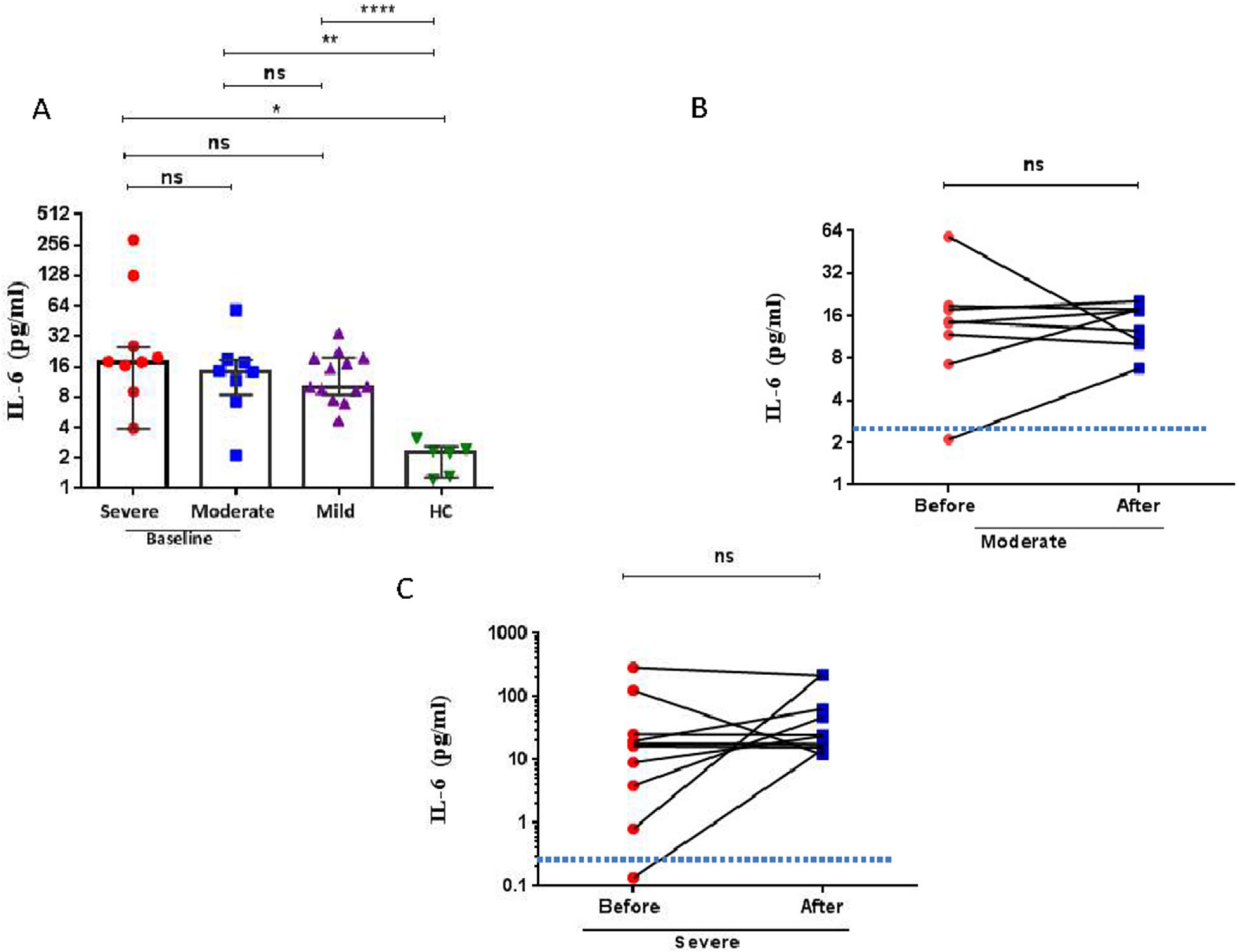
Serum interleukin (IL)-6 levels in COVID-19 patients. (A) Dot blots of individual values and median (5–95% percentiles) of serum IL-6 levels before remdisivir and dexamethasone therapy in severe and moderate COVID-19 patients compared with levels in mild patients and healthy control (HC) subjects (B) The comparison of IL-6 levels before and after 5–7 days remdisivir and dexamethasone therapy in moderate COVID-19 patients. (C) The comparison of IL-6 levels before and after 5–7 days remdisivir and dexamethasone therapy in severe COVID-19 patients. A Mann-Whitney non-parametric test was used to analyse data between groups and a paired Wilcoxon non-parametric test was used for the before and after treatment comparison ∗p < 0.05, ∗∗p < 0.01, ∗∗∗p < 0.001, ∗∗∗∗p < 0.0001, ns; non-significant. The dotted line in (B) and (C) represents the mean level of IL-6 reported in healthy control subjects.

As a marker of the cytokine storm, serum levels of IL-6 and IL-8 were evaluated in the COVID-19 groups and HC at baseline and in the moderate and severe COVID-19 patients over 5–7 days of treatment with remdesivir and dexamethasone. Baseline levels of serum IL-6 were significantly elevated in all COVID-19 patients compared with HC and there was no difference observed in these levels in association with severity. In addition, there was no significant change in the levels of these cytokines before and after treatment for any severity group (Figure 2A-C, Tables 3 & 4).

**Figure 3:**
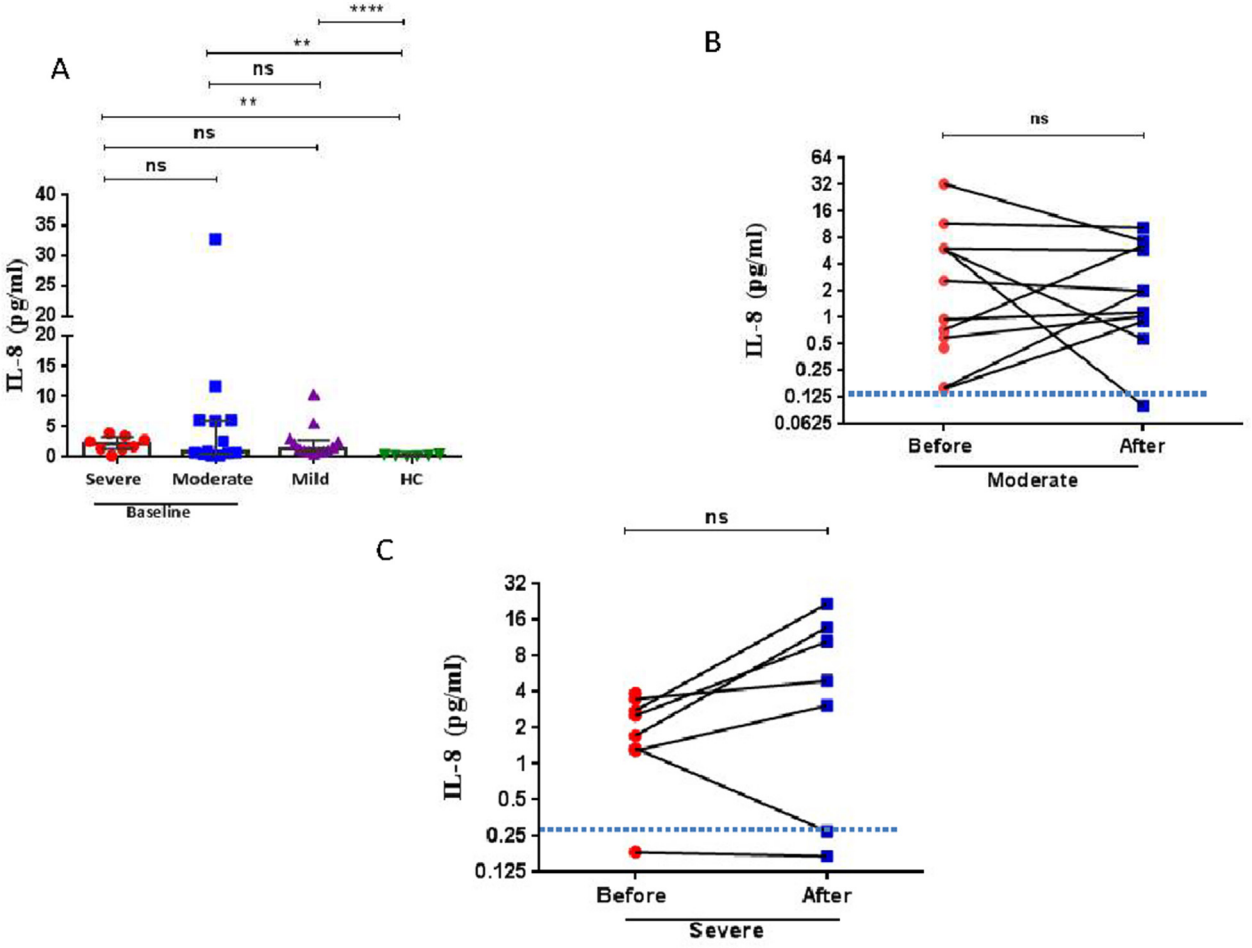
Serum interleukin (IL)-8 levels in COVID-19 patients. (A) Dot blots of individual values and median (5–95% percentiles) of serum IL-6 levels before remdisivir and dexamethasone therapy in severe and moderate COVID-19 patients compared with levels in mild patients and healthy control (HC) subjects. (B) The comparison of IL-8 levels before and after 5–7 days remdisivir and dexamethasone therapy in moderate COVID-19 patients. (C) The comparison of IL-8 levels before and after 5–7 days remdisivir and dexamethasone therapy in severe COVID-19 patients. A Mann-Whitney non-parametric test was used to analyse data between groups and a paired Wilcoxon non-parametric test was used for the before and after treatment comparison ∗p < 0.05, ∗∗p < 0.01, ∗∗∗∗p < 0.0001 ns; non-significant. The dotted line in (B) and (C) represents the mean level of IL-8 reported in healthy control subjects.

Serum IL-8 levels were also significantly elevated in COVID-19 patients compared to HC at baseline (Figure 3A, Tables 3 & 4). There was no significant change in the levels of IL-8 before and after treatment for any severity group (Figure 3B, C, Tables 3 & 4).

### 3.4 Effect of sex and age on serum ACE2 levels

The serum sACE2 levels were analysed according to sex in moderate and severe COVID-19 patients at the baseline and after treatment. Females having higher levels than males and there was significant difference in serum sACE2 levels between the baseline and after treatment in moderate (F vs M: P = 0.0260) and severe (F vs M: P = 0.0022) COVID-19 patients (data not shown).

There were no significant correlations between serum sACE2 levels with age in moderate patients before treatment (r = 0.0239, p = 0.9594), after treatment (r = 0.4462, p = 0.3156) or in severe COVID-19 patients before (r = 0.0507, p = 0.9240) or after treatment (r = 0.4173, p = 0.4104) (data not shown).

## 4 Discussion

We demonstrated that sACE2 levels are lower in severe and moderate COVID-19 patients compared to mild subjects at admission. Over 5–7 days as patients recovered, sACE2 levels were enhanced in all moderate patients but only in half of the patients with severe disease. This reflects the observation sACE2 levels increased in female but not male severe patients. In contrast, although serum cytokines were elevated in all COVID-19 subjects, the levels were unchanged over the following 5–7 days. The levels of LDH in the severe COVID-19 patients increased over the duration of hospitalization which may reflect ongoing cell death in response to the host defence to infection. In addition, the elevated levels of CRP seen in moderate and severe patients was reduced over time/with therapy. In addition, IL-6 and IL-8 levels were significantly higher in COVID-19 patients as compared to HC but no difference was observed in these levels in association with severity. Moreover, there was no significant change in the levels of IL-6 and IL-8 before and after treatment for any severity group. These data highlight the dissociation between sACE2 and serum cytokine levels at baseline and during recovery over time.

Early diagnosis and suitable clinical monitoring and management of COVID-19 patients is critical in preventing severe consequences and even death in hospitalised COVID-19 patients [23]. Multiple efforts are underway globally to inhibit virus infection to control SARS-COV-2 infection and the COVID-19 pandemic. Until global vaccination programmes achieve suitable levels, additional approaches such as blocking viral entry using transmembrane protease serine 2 inhibitors; sACE2 or antibodies, and viral RNA-dependent RNA polymerase (eg, remdesivir) may be useful [23].

ACE2 receptors plays an important role in the binding of SARS-CoV-2 and is located on the surface of many cells including respiratory epithelial cells. The ACE2 receptor enables entry of the virus into target cells and vaccines specifically targeting the viral spike protein–ACE2 interaction are extremely beneficial clinically [24]. Therefore, ACE2 is at the core of COVID-19 research and drug development.

The enhanced levels of sACE2 levels seen over time following treatment compared to the low levels seen at baseline may reflect a natural response or result from intervention with remdesivir and dexamethasone therapy. This raises the question as to the clinical relevance of low sACE2 in moderate and severe patients and the restoration of ‘normal’ levels over time during recovery. This may reflect shedding of ACE2 following binding by SARS-CoV-2. Haga and colleagues demonstrated that binding of recombinant and virion-associated SARS Spike (S) protein to ACE2 receptors induces ACE2 shedding in a disintegrin and metalloproteinase domain 17 (ADAM17)/tumor necrosis factor alpha (TNF-α)-converting enzyme (TACE)-dependent manner [24, 25]. Moreover, ACE2 shedding might reduce ACE2 surface receptor expression. Exposure of HEK293 cells to the phorbol ester phorbol myristate acetate (PMA) *in vitro* induced ACE2 shedding and reduced the level of cell-associated ACE2 receptors [25, 26]. Furthermore, incubation of cells with control virus-like particles (VLPs) which do not contain any viral glycoprotein does not induce ACE2 shedding whereas VLPs bearing the SARS-S protein triggers ACE2 shedding. Thus, SARS-CoV-2 can downregulate its receptor and the S protein might be critical to receptor interference [27].

A previous study reported no significant difference in serum levels of sACE2 between individuals who were PCR-positive or -negative for SARS-CoV-2 infection [28]. That study, however, did not examine serum levels of sACE2 in patients with differing severity of disease nor the effect of antiviral and/or dexamethasone treatment *per se*. Injection of exogenous human recombinant soluble (hrs)ACE2 in COVID-19 patients has beneficial effects [8, 17, 29, 30]. Interestingly, 9 days after the onset of symptoms, intravenous infusion with hrsACE2 (APN01; 0.4 mg/kg) was successful in treating patients [30]. Importantly, hrsACE2 treatment did not interfere with the generation of neutralising antibodies. The authors suggested that hrsACE2 could block the systemic spread of the virus from the lung to other organs [30]. One previous study has demonstrated a significant increase in lung endothelial cell immunostaining for ACE2 and for serum ACE2 levels in 15 COVID-19 patients with ARDS and 13 non-COVID-19 patients with ARDS compared to control tissues from patients who had a lobectomy for lung cancer [31]. This suggests that differences in sACE2 may reflect the presence of COVID-19-induced ARDS, which reversed over time/with treatment. However, in our study the CT and APACHE II scores were significantly greater in the severe patients compared with moderate patients whilst sACE2 levels were similar. Future studies are required in a larger multi-centre cohort to determine the precise link between ARDS and sACE2 levels.

An age- and sex-relationship between ACE2 in humans exists. sACE2 levels are low in both sexes up to the age of 12 whereupon it increases to a greater extent in males such that males older than 15 have higher sACE2 levels than women [32]. In our study, we found no relationship between sACE2 and age but did report a significant difference between sexes at baseline in sACE2 levels. This difference was lost over time/with treatment.

Higher levels of sACE2 are associated with a greater risk for severe COVID-19 [33] which may explain the greater susceptibility to infection by SARS-CoV-2. Smoking and individuals with Type 2 diabetes and/or obesity also have higher serum sACE2 levels [34]. However, plasma concentrations of sACE2 are much lower in diabetics with chronic disease than in healthy controls and are further reduced by the use of hypoglycemia drugs. This may provide a rationale for why diabetics with lower plasma levels of sACE2 may be more susceptible to severe COVID-19 [35].

Serum levels of circulating sACE2 should be considered as a potential interface between chronic inflammation, cardiovascular disease and COVID-19 susceptibility [36]. Elderly patients with aortic stenosis have markedly elevated serum ACE2 levels together with altered left and right ventricular functions, which may pose higher risks during COVID-19 [36]. In addition, high plasma sACE2 levels may be a predictor of COVID-19 severity and outcomes as well as a risk factor for hypertension, pre-existing heart disease and pre-existing kidney disease [37]. In contrast, in lung diseases circulating sACE2 levels were significantly reduced in COPD and pulmonary fibrosis (PF) compared to healthy control [38]. sACE2 levels may merely reflect the inflammatory state which is indicated by serum CRP levels. CRP levels at baseline were significantly higher in the severe and moderate COVID-19 patients compared to those with mild disease and significantly lowered with time/treatment. It is possible, therefore, that as sACE2 levels track inversely with CRP levels that there is a causal effect. This requires further investigation.

Thus, age and co-morbidities may be important confounders, but it is unlikely that the age of the patients is a confounding factor in this study since all of participants are adults of a similar age. Furthermore, there was no link between sACE2 levels and any underlying diabetes, smoking and hypertension in severe and moderate COVID-19 patients in this study (data not shown).

Despite the changes observed in sACE2 levels we did not find any significant differences in serum IL-6 or IL-8 levels over the duration of the study. Many studies [39, 40] report that mild patients have lower IL-6 and IL-8 levels as compared to moderate and severe patients and severe patients have higher levels of IL-6 and IL-8 as compared to moderate patients. Although we did not find a significant difference in cytokine levels between patients with mild, moderate and severe forms of the disease, there is trend towards an increase in these levels between groups. This may reflect the low number in our study but may also reflect other factors such as the precise timing of sample collection after infection and/or hospitalization. In addition, the timing and/or dose of the dexamethasone treatment in these patients or potential ethnic differences between patients recruited here and in other studies may affect the results.

Although there are several strengths to our study we recognize that there are some limitations. These include the low number of participants, having only males in the healthy control group is a limitation of the study as is the collection of samples from only subsets of recruited patients rather than all subjects. In addition, we do not have untreated, severe COVID-19 subjects to act as controls as this would not be allowed by our Ethics Committee. Although we measured IL-6 and IL-8 levels, the cytokine storm involves many more cytokines [40]. A more complete picture of the changes in the cytokine storm following time/treatment using multiplex analysis should be undertaken in future studies.

In conclusion, whether the increased levels of sACE2 in the serum are a direct result of the antiviral and dexamethasone therapy or results from a normal compensatory mechanism over time to control the virus remains unknown as it was unethical to have a control untreated group of patients. The potential link with ARDS should be examined further in Iranian patients together with longitudinal follow-up of clinical outcomes. These changes in sACE2 in severe patients are independent of any anti-inflammatory effect of therapy as evidenced by unaltered cytokine levels and highlights the potential for direct targeting of ACE2 in treating severe COVID-19.

## Declarations

### Author contribution statement

Esmaeil Mortaz: Conceived and designed the experiments; Performed the experiments; Analyzed and interpreted the data; Wrote the paper.

Neda Dalil Roofchayee: Analyzed and interpreted the data; Contributed reagents, materials, analysis tools or data; Wrote the paper.

Hamidreza Jamaati, Hakime Sheikhzade, Maryam Mirenayat, Mohsen Sadeghi and Somayeh Lookzadeh: Performed the experiments; Analyzed and interpreted the data; Contributed reagents, materials, analysis tools or data.

Neda K Dezfuli: Contributed reagents, materials, analysis tools or data.

Gert Folkerts, Sharon Mumby, Johan Garssen and Ian M. Adcock: Conceived and designed the experiments; Analyzed and interpreted the data; Wrote the paper.

### Funding statement

Ian M. Adcock was supported by the Welcome Trust; (093080/Z/10/Z), the EPSRC (EP/T003189/1), the Community Jameel Imperial College COVID-19 Excellence Fund (G26290) and by the UK MRC (MR/T010371/1). Sharon Mumby was supported by EU project 853850.

### Data availability statement

Data included in article/supplementary material/referenced in article.

### Declaration of interests statement

The authors declare no conflict of interest.

### Additional information

No additional information is available for this paper.

## References

[1] Yang X., Yu Y., Xu J., Shu H., Liu H., Wu Y. (2020). Clinical course and outcomes of critically ill patients with SARS-CoV-2 pneumonia in Wuhan, China: a single-centered, retrospective, observational study. Lancet Respir. Med..

[2] Zheng M., Gao Y., Wang G., Song G., Liu S., Sun D. (2020). Functional exhaustion of antiviral lymphocytes in COVID-19 patients. Cell. Mol. Immunol..

[3] Yuki K., Fujiogi M., Koutsogiannaki S. (2020).

[4] Mortaz E., Tabarsi P., Varahram M., Folkerts G., Adcock I.M. (2020). The immune response and immunopathology of COVID-19. Front. Immunol..

[5] Archer S.L., Sharp W.W., Weir E.K. (2020). Differentiating COVID-19 pneumonia from acute respiratory distress syndrome and high altitude pulmonary edema: therapeutic implications. Circulation.

[6] Imai Y., Kuba K., Penninger J.M. (2006). The renin–angiotensin system in acute respiratory distress syndrome. Drug Discov. Today Dis. Mech..

[7] Imai Y., Kuba K., Penninger J.M. (2007). Angiotensin-converting enzyme 2 in acute respiratory distress syndrome. Cell. Mol. Life Sci..

[8] Monteil V., Kwon H., Prado P., Hagelkrüys A., Wimmer R.A., Stahl M. (2020). Inhibition of SARS-CoV-2 infections in engineered human tissues using clinical-grade soluble human ACE2. Cell.

[9] Graham R., Baric R., Li F. (2020). Receptor Recognition by the Novel Coronavirus from Wuhan:An analysis based on decade-long structural studies of SARS 3 2020. J. Virol..

[10] Cuba K., Imai Y., Rao S., Gao H., Guo F. (2005). Guan Bet al. Un papel crucial de la enzima convertidora de angiotensina 2 (ACE2) en la lesión pulmonar inducida por coronavirus del SARS. Nat. Med..

[11] Tipnis S.R., Hooper N.M., Hyde R., Karran E., Christie G., Turner A.J. (2000). A human homolog of angiotensin-converting enzyme: cloning and functional expression as a captopril-insensitive carboxypeptidase. J. Biol. Chem..

[12] Samavati L., Uhal B.D. (2020). ACE2, much more than just a receptor for SARS-COV-2. Front. Cell. Inf. Microbiol..

[13] Yeung M.L., Teng J.L.L., Jia L., Zhang C., Huang C., Cai J.-P. (2021). Soluble ACE2-mediated cell entry of SARS-CoV-2 via interaction with proteins related to the renin-angiotensin system. Cell.

[14] Iwasaki M., Saito J., Zhao H., Sakamoto A., Hirota K., Ma D. (2021). Inflammation triggered by sars-cov-2 and ace2 augment drives multiple organ failure of severe covid-19: molecular mechanisms and implications. Inflammation.

[15] Zhang H., Penninger J.M., Li Y., Zhong N., Slutsky A.S. (2020). Angiotensin-converting enzyme 2 (ACE2) as a SARS-CoV-2 receptor: molecular mechanisms and potential therapeutic target. Intensive Care Med..

[16] Chan K.K., Dorosky D., Sharma P., Abbasi S.A., Dye J.M., Kranz D.M. (2020). Engineering human ACE2 to optimize binding to the spike protein of SARS coronavirus 2. Science.

[17] Abd El-Aziz T.M., Al-Sabi A., Stockand J.D. (2020). Human recombinant soluble ACE2 (hrsACE2) shows promise for treating severe COVID19. Signal Transd. Targ. Therap..

[18] World Health O. (2020). Clinical management of severe acute respiratory infection (SARI) when COVID-19 disease is suspected. Interim guidance. Pediatria i Medycyna Rodzinna.

[19] Díez-Pérez A., Sabaté R.A., Petit I., Brasé A., Horcajada J.P., Güerri-Fernández R. (2021).

[20] Gandhi R., Lynch J., Del Rio C. (2020). Mild or moderate COVID-19. N. Engl. J. Med..

[21] Rahmanzade R., Rahmanzadeh R., Hashemian S.M., Tabarsi P. (2020). Iran's approach to COVID-19: evolving treatment protocols and ongoing clinical trials. Front. Public Health.

[22] Horby P.W., Campbell M., Staplin N., Spata E., Emberson J.R., Pessoa-Amorim G. (2021). Tocilizumab in patients admitted to hospital with COVID-19 (RECOVERY): preliminary results of a randomised, controlled, open-label, platform trial. J. Am. Med. Dir. Assoc..

[23] Borges do Nascimento I.J., von Groote T.C., O’Mathúna D.P., Abdulazeem H.M., Henderson C., Jayarajah U. (2020). Clinical, laboratory and radiological characteristics and outcomes of novel coronavirus (SARS-CoV-2) infection in humans: a systematic review and series of meta-analyses. PLoS One.

[24] Corey L., Mascola J.R., Fauci A.S., Collins F.S. (2020). A strategic approach to COVID-19 vaccine R&D. Science.

[25] Lambert D.W., Yarski M., Warner F.J., Thornhill P., Parkin E.T., Smith A.I. (2005). Tumor necrosis factor-α convertase (ADAM17) mediates regulated ectodomain shedding of the severe-acute respiratory syndrome-coronavirus (SARS-CoV) receptor, angiotensin-converting enzyme-2 (ACE2). J. Biol. Chem..

[26] Haga S., Yamamoto N., Nakai-Murakami C., Osawa Y., Tokunaga K., Sata T. (2008). Modulation of TNF-α-converting enzyme by the spike protein of SARS-CoV and ACE2 induces TNF-α production and facilitates viral entry. Proc. Natl. Acad. Sci. Unit. States Am..

[27] Glowacka I., Bertram S., Herzog P., Pfefferle S., Steffen I., Muench M.O. (2010). Differential downregulation of ACE2 by the spike proteins of severe acute respiratory syndrome coronavirus and human coronavirus NL63. J. Virol..

[28] Rieder M., Wirth L., Pollmeier L., Jeserich M., Goller I., Baldus N. (2021). Serum ACE2, angiotensin II, and aldosterone levels are unchanged in patients with COVID-19. Am. J. Hypertens..

[29] Mostafa-Hedeab G. (2020). ACE2 as drug target of COVID-19 virus treatment, simplified updated review. Rep. Biochem. Mol. Biol..

[30] Zoufaly A., Poglitsch M., Aberle J.H., Hoepler W., Seitz T., Traugott M. (2020). Human recombinant soluble ACE2 in severe COVID-19. Lancet Respir. Med..

[31] Gerard L., Lecocq M., Bouzin C., Hoton D., Schmit G., Pereira J.P. (2021). Increased angiotensin-converting enzyme 2 and loss of alveolar type II cells in COVID-19-related acute respiratory distress syndrome. Am. J. Respir. Crit. Care Med..

[32] Swärd P., Edsfeldt A., Reepalu A., Jehpsson L., Rosengren B.E., Karlsson M.K. (2020). Age and sex differences in soluble ACE2 may give insights for COVID-19. Crit. Care.

[33] Grasselli G., Zangrillo A., Zanella A., Antonelli M., Cabrini L., Castelli A. (2020). Baseline characteristics and outcomes of 1591 patients infected with SARS-CoV-2 admitted to ICUs of the Lombardy Region. Italy. Jama..

[34] Emilsson V., Gudmundsson E.F., Aspelund T., Jonsson B.G., Gudjonsson A., Launer L.J. (2021). Serum levels of ACE2 are higher in patients with obesity and diabetes. Obes Sci Pract.

[35] Zhang Y., Sun Y., Liu K., Alolga R.N., Xu X., Feng G. (2021). Low plasma angiotensin-converting enzyme 2 level in diabetics increases the risk of severe COVID-19 infection. Aging.

[36] Fagyas M., Kertész A., Siket I.M., Bánhegyi V., Kracskó B., Szegedi A. (2021). Level of the SARS-CoV-2 receptor ACE2 activity is highly elevated in old-aged patients with aortic stenosis: implications for ACE2 as a biomarker for the severity of COVID-19. GeroScience.

[37] Kragstrup T.W., Singh H.S., Grundberg I., Nielsen A.L.-L., Rivellese F., Mehta A. (2021). Plasma ACE2 predicts outcome of COVID-19 in hospitalized patients. PLoS One.

[38] Fließer E., Birnhuber A., Marsh L.M., Gschwandtner E., Klepetko W., Olschewski H. (2021). Dysbalance of ACE2 levels–a possible cause for severe COVID-19 outcome in COPD. J. Pathol Clin. Res..

[39] Del Valle D.M., Kim-Schulze S., Huang H.H., Beckmann N.D., Nirenberg S., Wang B. (2020). An inflammatory cytokine signature predicts COVID-19 severity and survival. Nat. Med..

[40] Dorgham K., Quentric P., Gökkaya M., Marot S., Parizot C., Sauce D. (2021). Distinct cytokine profiles associated with COVID-19 severity and mortality. J. Allergy Clin. Immunol..

